# Supplementary material for: Neutrophil Extracellular Traps Promote Metastases of Colorectal Cancers through Activation of ERK Signaling by Releasing Neutrophil Elastase
Source: Int J Mol Sci. 2023 Jan 6;24(2):1118. doi: 10.3390/ijms24021118 (PMC9867023; doi:10.3390/ijms24021118)
Supplement: Supplementary file 1 [file ijms-24-01118-s001.zip › Supplementaly tables and figures.pdf]

Table S1. Correlation between Cit-H3 expression and Clinicopathological characteristics in 133 CRC patients.

		Cit-H3 expression		
variables		Low (n=66)	High (n=67)	*P*
Age,y	Mean±SD	68.4±10.3	69.1±9.5	0.69
Gender	Male	42	44	0.81
	Female	24	23	
Location	Colon	39	45	0.43
	Rectum	27	22	
Histology	tub1/tub2	62	61	0.53
	others	4	6	
T factor	Tis,T1,T2	20	17	0.85
	T3,T4	46	50	
N factor	negative	46	40	0.23
	positive	20	27	
Preoperative therapy	(+)	4	4	0.98
	(-)	62	63	
Adjuvant therapy	(+)	26	26	0.94
	(-)	40	41	
Lymphatic invasion	negative	41	39	0.65
	positive	25	28	
Venous invasion	negative	31	30	0.8
	positive	35	37	
NLR	＜3	46	45	0.75
	≧3	20	22	
CEA levels (ng/mL)	＜5	38	41	0.67
	≧5	28	26	
CA19-9 levels (U/mL)	＜37	58	61	0.55
	≧37	8	6	

Table S2. Univariate and multivariate analysis using clinicopathological characteristics and Cit-H3 expression for OS in 133 CRC patients

	univariate analysis			multivariate analysis		
**variables**	**HR**	**95%CI**	***P***	**HR**	**95%CI**	***P***
Age,years (< 68 vs. ≥ 68)	2.09	0.86 - 5.08	0.11			
Sex (Male vs. Female)	0.63	0.25 - 1.60	0.33			
Location (Colon vs. Rectum)	1.76	0.78 - 3.99	0.18			
T factor (Tis - T2 vs. T3 - T4)	1.38	0.51 - 3.71	0.53			
N factor (negative vs. positive)	1.44	0.63 - 3.27	0.39			
Lymphatic invasion (ly0 vs. ≥ ly1)	2.03	0.89 - 4.64	0.09	1.73	0.75 - 3.99	0.2
Venous invasion (v0 vs. ≥ v1)	0.96	0.42 - 2.18	0.92			
Preoperative therapy (no vs. yes)	4.46	1.65 - 12.03	0.0032	3.94	1.45 - 10.86	0.0073
Adjuvant therapy (no vs. yes)	0.60	0.24 – 1.46	0.26			
NLR (< 3 vs. ≥ 3)	0.83	0.33 - 2.12	0.7			
CEA (ng/mL, < 5 vs. ≥ 5)	1.22	0.53 - 2.77	0.64			
CA19-9 (U/mL, < 37 vs. ≥ 37)	1.34	0.40 - 4.51	0.64			
Cit-H3 expression (low vs. high)	2.03	0.86 - 4.88	0.1	1.97	0.83 - 4.66	0.12

Table S3. Correlation between serum MPO-DNA levels and Clinicopathological characteristics in 67 CRC patients.

		serum MPO-DNA levels		
variables		Low (n=36)	High (n=31)	*P*
Age,y	Mean±SD	69±14.2	67.4±14.4	0.65
Gender	Male	20	15	0.37
	Female	16	16	
Location	Colon	19	24	
	Rectum	17	7	0.03
Histology	tub1/tub2	32	23	
	others	4	8	0.11
T factor	Tis-T3	30	25	
	T4	6	6	0.78
N factor	negative	19	17	
	positive	17	14	0.86
Preorerative therapy	(+)	6	2	
	(-)	34	29	0.18
Adjuvant therapy	(+)	19	16	0.92
	(-)	17	15	
Lymphatic invasion	negative	30	27	0.67
	positive	6	4	
Venous invasion	negative	10	13	0.22
	positive	26	18	
NLR	＜3	24	19	0.64
	≧3	12	12	
CEA levels (ng/mL)	＜5	21	20	0.45
	≧5	16	11	
CA19-9 levels (U/mL)	＜37	32	27	0.82
	≧37	4	4	

Table S4. Univariate and multivariate analysis using clinicopathological characteristics and serum MPO-DNA levels for OS in 67 CRC patients

	univariate analysis			multivariate analysis		
variables	HR	95%CI	*P*	HR	95%CI	*P*
Age,years (< 68 vs. ≥ 68)	2.14	0.47 - 9.72	0.33			
Sex (Male vs. Female)	0.45	0.14 - 1.50	0.2			
Location (Colon vs. Rectum)	0.13	0.017 - 1.04	0.05	0.11	0.014 - 0.89	0.038
T factor (Tis - T3 vs. T4)	1.2	0.60 - 2.37	0.61			
N factor (negative vs. positive)	0.84	0.27 - 2.65	0.77			
Lymphatic invasion (ly0 vs. ≥ ly1)	1.26	0.28 - 5.78	0.76			
Venous invasion (v0 vs. ≥ v1)	1.14	0.34 - 3.79	0.83			
Preoperative therapy (no vs. yes)	0.64	0.08 - 4.97	0.67			
Adjuvant therapy (no vs. yes)	0.62	0.19 - 1.95	0.41			
NLR (< 3 vs. ≥ 3)	1.33	0.42 - 4.19	0.63			
CEA (ng/mL, < 5 vs. ≥ 5)	1.15	0.37 - 3.63	0.81			
CA19-9 (U/mL, < 37 vs. ≥ 37)	15.03	4.59 - 49.20	< 0.0001	18.63	1.13 - 71.27	< 0.0001
MPO-DNA levels (low vs. high)	1.68	0.53 - 5.29	0.38			

Table S5. List of antibodies used for IHC

Antibody	Host	Company	dilution ratio
anti-cit H3	rabbit	abcam	1:300
anti-MPO	mouse	Leica	ready to use
anti-MMP9	mouse	Santa Cruz	1:100
anti-Neutrophil Elastase	mouse	Santa Cruz	1:100
anti-Ki-67	mouse	DAKO	1:100

MMP9, matrix metalloproteinase 9

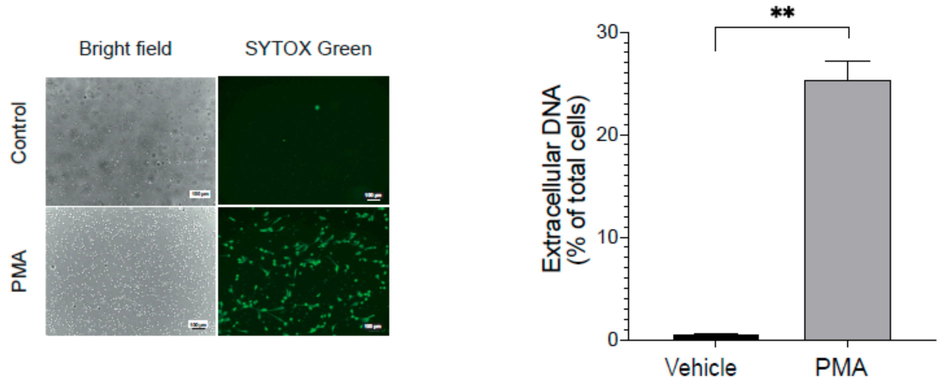

Figure S1.

Human neutrophils were treated with phorbol myristate acetate (PMA) in order to induce neutrophil extracellular traps (NETs). NETs were confirmed by extracellular DNA staining using SYTOX green. The percentage of SYTOX green positive cells per 5 high power fields were recorded. Mean; bars, ±SEM, n = 3. $^{**}P < 0.01$ by Student's t test. Scale bar = 100 μm

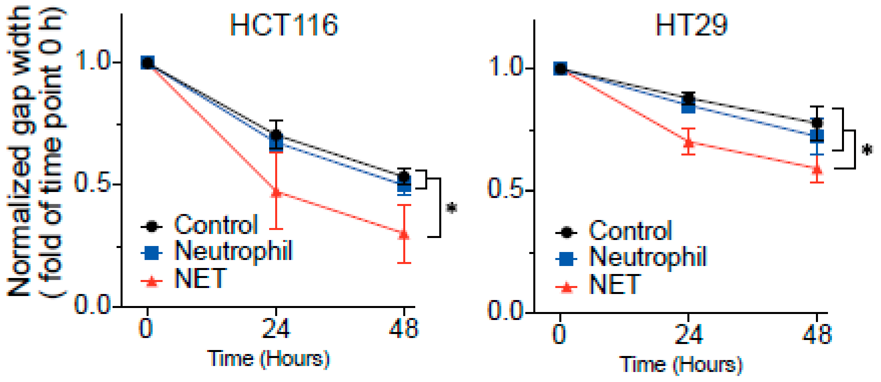

Figure S2.

Cell migration assay using scratch wound healing. HCT116 and HT29 cells were incubated with NET conditioned medium or neutrophil control medium. All dishes were analyzed at 0, 24, 48 or 72 hours. Normalize wounding area at 0h as 1. Mean; bars, ±SD, n = 6. $^{*}P < 0.05$ by Student's t test.

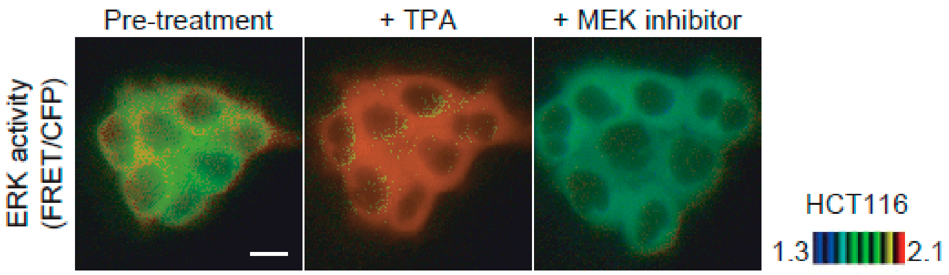

Figura S3 and video S1.

FRET-based live cell imaging using the biosensor of ERK (FRET/CFP) in HCT116 cells. HCT116 cells were serum-starved for 6 h. Cells were treated with 10 nM of MEK inhibitor 30minuite after treatment with 100 nM of 12-O-tetradecanoylphorbol-13-acetate (TPA). Representative FRET/CFP ratio movie and images of pre-/post-treatmentat are shown in the intensity-modulated display mode. Scale bar = 10 μm.
